# Evaluating High Mobility Group Box 1 (HMGB1) as a prognostic biomarker for mortality in canine systemic inflammatory response syndrome

**DOI:** 10.18849/ve.v11i3.741

**Published:** 2026-09-21

**Authors:** Hannah Abrahams

**Affiliations:** The University of Sydney Australia

**Keywords:** CANINE, HMGB1 PROTEIN, HIGH MOBILITY GROUP BOX 1, MORTALITY, PROGNOSIS, SIRS

## Abstract

**PICO question:**

In canine patients with infectious systemic inflammatory response syndrome (SIRS), how do increased levels of High Mobility Group Box 1 (HMGB1) protein, compared to low levels, relate to mortality outcomes?

**Clinical bottom line:**

**Category of research:**

Prognosis.

**Number and type of study designs reviewed:**

Three studies were critically reviewed. All three studies were prospective observational studies.

**Strength of evidence:**

Weak.

**Outcomes reported:**

All three studies included in this Knowledge Summary evaluated canine patients with SIRS, encompassing both infectious and non-infectious causes. The second and third paper both found a significant association between elevated serum HMGB1 levels and increased mortality in dogs with SIRS. The first paper, while focusing specifically on infectious SIRS, did not find a statistically significant correlation between HMGB1 levels and mortality, although HMGB1 levels were elevated in SIRS cases compared to healthy controls. Variability in the timing of HMGB1 measurements, along with differences in study design, population heterogeneity, and small sample sizes, may have contributed to inconsistencies in the findings. Overall, the evidence suggests a potential association between higher HMGB1 levels and increased mortality in dogs with SIRS, but further standardised research is needed to strengthen this conclusion.

**Conclusion:**

While two studies identified an association between elevated HMGB1 and mortality, the findings are inconclusive. The strength of evidence is restricted by diagnostic and methodological limitations, making for weak quality evidence overall. Future research should prioritise standardised measurement timing, larger and more homogeneous populations, and explore HMGB1’s role in disease progression to fully establish its utility as a prognostic marker in canine SIRS.

How to apply this evidence in practice

The application of evidence into practice should take into account multiple factors, not limited to: individual clinical expertise, patient’s circumstances and owners’ values, country, location or clinic where you work, the individual case in front of you, the availability of therapies and resources.

Knowledge Summaries are a resource to help reinforce or inform decision making. They do not override the responsibility or judgement of the practitioner to do what is best for the animal in their care.

## The evidence

Three studies evaluated the role of High Mobility Group Box 1 (HMGB1) in systemic inflammatory response syndrome pathophysiology and its association with mortality in canine patients (Goggs & Letendre, 2019; Ishida et al., 2011; Yu et al., 2010).

Yu et al. (2010) conducted a prospective cohort study comparing high serum HMGB1 levels in SIRS patients with mortality outcomes. The study found that HMGB1 levels were significantly higher in non-survivors compared to survivors, highlighting HMGB1 as a potential mortality marker. The evidence in this study is of moderate quality based on its statistical association and methodology, though small sample size and lack of differentiation between infectious and non-infectious SIRS cases weaken its strength.

Another prospective cohort study by Ishida et al. (2011) also identified a significant correlation between elevated HMGB1 levels and increased mortality. This study had the largest sample size and included a diverse range of diseases, but it did not categorise cases as infectious or non-infectious, limiting interpretation regarding HMGB1’s role in infectious SIRS specifically. The evidence quality is moderate, supported by the larger cohort, though methodological clarity was lacking, and no details were provided about the size or direction of associations between subgroups.

In contrast, Goggs & Letendre (2019) performed a prospective case-control study focused specifically on infectious SIRS and found no significant association between HMGB1 levels and mortality. The evidence quality is weak due to the study design limiting causal inference and the unclear relationship between HMGB1, C-reactive protein (CRP), and disease progression. Additionally, the timing of HMGB1 measurement was not standardised across these studies, which may have influenced the observed correlations with survival. In canine patients with infectious systemic inflammatory response syndrome, elevated HMGB1 concentrations may be associated with mortality, although current evidence is insufficient to establish HMGB1 as a reliable prognostic biomarker.

## Summary of the evidence

**Goggs & Letendre (2019):** Evaluation of the host cytokine response in dogs with sepsis and non-infectious systemic inflammatory response syndrome **Aim: **To evaluate the host cytokine response, including HMGB1, in dogs with sepsis and non-infectious SIRS and assess associations with survival.

Population:	Dogs weighing ≥ 3 kg enrolled at a university teaching hospital between June 2015 and February 2016. The study was conducted at Cornell University, Ithaca, New York USA.
Sample size:	70 dogs.
Intervention details:	The dogs were grouped into a healthy control group (15/70), a non-infectious SIRS group (10/70) and an infectious SIRS group (45/70). The infectious SIRS group contained dogs that satisfied ≥ 2 SIRS criteria and had a documented positive bacterial infection. The non-infectious SIRS group contained dogs that satisfied ≥ 2 SIRS criteria but had no evidence of infection, as well as dogs for which bacterial infection was initially suspected but not subsequently confirmed, and for which an alternative non-infectious diagnosis was established. Dogs < 3 kg or with documented coagulopathy were excluded. Clinical and laboratory data were collected at enrolment, including blood pressure, pulse oximetry, physical examination findings, complete blood count, serum biochemistry, and blood lactate concentration. Plasma HMGB1 concentrations were measured using a commercial enzyme-linked immunosorbent assay (ELISA). Plasma concentrations of multiple inflammatory cytokines were also measured. Patients were followed to hospital discharge, with outcome recorded as survival, death, or euthanasia due to disease severity.
Study design:	Prospective observational case-control study.
Outcome Studied:	To compare plasma HMGB1 concentrations between dogs with sepsis, non-infectious SIRS and healthy controls, and to assess the association between HMGB1 concentrations and survival to hospital discharge.
Main Findings (relevant to PICO question):	Plasma HMGB1 concentrations were significantly higher in dogs with sepsis than in healthy controls. There was no significant difference in HMGB1 concentration between dogs with sepsis and those with non-infectious SIRS (P = 1.000). Among dogs with SIRS, HMGB1 concentration did not differ significantly between survivors and non-survivors (P = 0.385). HMGB1 was therefore associated with the presence of sepsis compared with healthy controls, but was not associated with survival to hospital discharge.
Limitations:	Small sample size and single-centre design limits the generalisability. Differences in disease type and severity among the population were not accounted for. The nonspecific nature of the SIRS criteria may have resulted in misclassification of dogs into the infectious and non-infectious SIRS groups. Differences in sample timing may affect results due to the kinetic nature of HMGB1. Treatment and outcome monitoring protocols were not standardised. Low disease severity in the study population may have limited the ability of HMGB1 to distinguish survivors from non-survivors. Animals were not screened for or excluded based on the use of antibiotics or anti-inflammatories prior to study enrolment. Financial restraints might have limited lab results reliability due to single measurements instead of repeated measurements. No specific criteria for euthanasia were mentioned – if decisions were subjective or owner-driven, this could have affected mortality rates. Nonspecific SIRS criteria may have led to misclassification between infectious and non-infectious groups.

**Ishida et al. (2011):** Plasma high-mobility group box 1 (HMGB1) in dogs with various diseases: comparison with C-reactive protein **Aim: **To evaluate plasma HMGB1 concentrations in dogs with various diseases and investigate their association with SIRS and clinical outcome.

Population:	Dogs referred to the Veterinary Medical Centre of the University of Tokyo (VMC-UT) between April and November 2008 were prospectively recruited. Clinically ill dogs meeting ≥ 2 criteria for systemic inflammatory response syndrome (SIRS) were included. Thirty-six clinically healthy Beagles owned by VMC-UT and used as blood donors served as controls.
Sample size:	343 dogs.
Intervention details:	The study population contained 307 clinically ill dogs and 36 clinically healthy Beagle dogs used as controls. 5 of the healthy control group underwent ovariohysterectomy and had blood collected before and at various time points following surgery. Clinically ill dogs were divided into ten groups based on their underlying conditions and further classified as either SIRS (133/307) or non-SIRS (174/307), according to whether they fulfilled at least two SIRS criteria as defined by Brady and Otto. The clinically ill group was also divided into survivors (101/307) and non-survivors (32/307) based on their mortality outcome. The study population contained 307 clinically ill dogs and 36 clinically healthy Beagle dogs used as controls. Blood samples were collected from all dogs, with plasma separated and stored at -20°C. Serum HMGB1 concentrations were measured in duplicate using a commercially available enzyme-linked immunosorbent assay (ELISA), with duplicate measurements averaged.
Study design:	Prospective observational case-control study.
Outcome Studied:	Plasma HMGB1 concentrations were measured at admission in healthy controls and clinically unwell dogs with and without SIRS. HMGB1 concentrations were compared between SIRS and non-SIRS dogs and between survivors and non-survivors within the SIRS group. C-reactive protein (CRP) concentrations were also measured in dogs with SIRS.
Main Findings (relevant to PICO question):	Dogs with SIRS had significantly higher plasma HMGB1 concentrations than dogs without SIRS (median 2.79 ng/mL, range 0–174.70 vs 0.25 ng/mL, range 0–78.49; P < 0.001). Within the SIRS group, non-survivors had significantly higher HMGB1 concentrations than survivors (median 7.1 ng/mL, range 0–174.70 vs 2.40 ng/mL, range 0–133.3; P < 0.01). Of the 133 dogs with SIRS, 101 survived to 14 days (76%). Survival was lower in dogs with HMGB1 concentrations above the reference range (55%, 17/31) than in those within the reference range (84%, 84/102; P < 0.01). There was no significant difference in CRP concentrations between SIRS and non-SIRS dogs (P = 0.081).
Limitations:	The relatively small sample sizes for certain disease categories reduce the statistical power to detect significant differences and increases the likelihood of Type II errors. Multiple comparisons across HMGB1 and CRP increase the risk of false positives. Confounders such as age, sex, breed, and concurrent treatments were not fully controlled for. Selection bias of the control group (clinically healthy Beagles) is not representative other breeds in the population. The cross-sectional design limits temporal changes and causality in the relationship between HMGB1 and CRP and the disease progression. It is not reported whether mortality includes euthanasia cases and the reasons. The paper studies HMGB1 from SIRS cases in general, not specifically infectious SIRS.

**Yu et al. (2010):** High-mobility group box-1 as a surrogate prognostic marker in dogs with systemic inflammatory response syndrome **Aim: **To investigate whether plasma HMGB1 concentrations could serve as a prognostic marker for mortality in dogs with SIRS.

Population:	Dogs hospitalised through the Department of Veterinary Internal Medicine, Chonbuk National University, Jeonju, Korea, between January 2007 and May 2009 were included. The study comprised 28 dogs with SIRS, including infectious and non-infectious causes.
Sample size:	28 dogs.
Intervention details:	Twenty-eight dogs met the study inclusion criteria, which required ≥2 SIRS criteria based on physical examination findings and blood results using Hauptman et al.'s criteria for SIRS diagnosis. Patients were grouped into non-survivors and survivors at the end of the study, with mortality defined as death or euthanasia due to poor prognosis, and survival defined as discharge from the hospital. Clinical history and baseline clinical and laboratory data were recorded. Blood samples were collected by venipuncture, and plasma was stored at -80 °C until analysis. Plasma interleukin-6 (IL-6), interleukin-10 (IL-10) and high mobility group box 1 (HMGB1) concentrations were measured using enzyme-linked immunosorbent assays (ELISA). Cytokine concentrations were compared with those from 12 clinically healthy Beagles aged 1–2 years, which served as a laboratory reference group.
Study design:	Prospective observational study.
Outcome Studied:	To assess whether plasma HMGB1 concentrations between survivors and non-survivors with both infectious and non-infectious SIRS.
Main Findings (relevant to PICO question):	HMGB1 concentration was a significant discriminator between survivors and non-survivors with both infectious and non-infectious SIRS. Median plasma HMGB1 concentration was significantly higher in non-survivors than survivors (P = 0.007). The HMGB1 ratio was also significantly higher in non-survivors (P = 0.024). The area under the curve for HMGB1 was 0.794 (95% CI: 0.631–0.958), compared with 0.761 (95% CI: 0.581–0.941) for the HMGB1 ratio.
Limitations:	The study included only 28 dogs, which limits the statistical power and generalisability of the findings. Lack of data regarding which cytokines were measured as well as the cytokine concentrations found in the control group of healthy (or non-SIRS) dogs. Variations in disease progressions and the timing of the blood sample collection relative to the onset of SIRS symptoms may have affected the accuracy and comparability of the results. The inclusion of a two-month-old in the reference range introduces puppy populations which may have differing inflammatory responses with respect to adult dogs. The lower detection limits for IL-6, IL-10, and HMGB1 assays may have resulted in some values being assigned default minimum values affecting the actual concentrations, particularly in cases where cytokine levels were near the detection threshold. Lack of multivariate analysis means that confounding variables may not have been accounted for. Selection bias of the control group (clinically healthy Beagles) is not representative of other breeds in the population. Survival was defined as discharge from the hospital, which might not fully capture long-term outcomes. Post-discharge follow-up would provide a more complete picture of the dogs' prognoses.

## Appraisal, application and reflection

Understanding systemic inflammatory response syndrome in veterinary medicine is crucial due to its significant impact on patient outcomes. Systemic inflammatory response syndrome, classified into infectious or non-infectious, is an exaggerated defence response to stress (Robertson & Coopersmith, 2006). It is typically managed with fluid resuscitation, antimicrobial therapy, and supportive care; however, the prognosis often remains poor despite these therapeutic strategies (Liu et al., 2016). Despite extensive research into inflammatory biomarkers, the prognostic utility of individual biomarkers in canine sepsis and systemic inflammation remains uncertain, highlighting the need for further evaluation of reliable prognostic markers (Song et al., 2012; Gaudette et al., 2023). High Mobility Group Box 1, a pro-inflammatory cytokine released by activated macrophages or necrotic cells, has been found to be associated with the pathophysiology of SIRS (Deng et al., 2022; Koo et al., 2020). In human medicine, HMGB1 is recognised as a prognostic marker for SIRS and various other inflammatory conditions (Hatada et al., 2005; Suda et al., 2006; Wang et al., 1999). Furthermore, studies in mice show that the release of HMGB1 initiates both local and systemic inflammation, leading to coagulopathy and organ damage (Lan et al., 2017). This evidence firmly establishes HMGB1’s role in regulating inflammation. Given this role, inhibiting HMGB1 secretion in critically ill people has been shown to reduce SIRS signs and mortality, indicating this protein may improve survival rates in the critically ill (Hotchkiss et al., 2013; Lan et al., 2017; Matsuura et al., 2023; Young et al., 2023). High Mobility Group Box 1 is therefore a valuable research target, with the potential to predict and change SIRS outcomes.

Three studies relevant to the PICO question were identified (Goggs & Letendre, 2019; Ishida et al., 2011; Yu et al., 2010), all of which were prospective cohort or case-control studies providing weak to moderate quality evidence.

Both Yu et al. (2010) and Ishida et al. (2011) reported that serum HMGB1 levels were significantly higher in non-survivors compared to survivors, suggesting a potential association between mortality in the SIRS cases and the high serum HMGB1 group. This supports the idea that HMGB1 may serve as a useful prognostic marker. In contrast, Goggs et al. (2019) found no significant association between HMGB1 levels and mortality. However, they did observe that HMGB1 concentrations were significantly higher in dogs with infectious SIRS compared to healthy controls.

Differences in measurement timing can affect the accuracy and comparability of studies on HMGB1 and SIRS. High Mobility Group Box 1 levels vary depending on when samples are collected postinjury or surgery (Milić et al., 2017). The lack of statistical significance between survivors and non-survivors in the Goggs & Letendre (2019) study could be due to these timing discrepancies and the kinetic fluctuations of HMGB1 in the blood. Additionally, laboratory methodology differences pose a further limitation. Although each study used commercially available human enzyme-linked immunosorbent assay kits to quantify HMGB1, these kits, commonly used in veterinary research, are not specifically validated for use in canine samples.

Validation is crucial and should assess accuracy, precision, linearity, detection limits, and establish appropriate reference ranges. Both Goggs & Letendre (2019) and Ishida et al. (2011) referenced previous validation studies; however, these studies were not included in the evidence appraisal undertaken for this Knowledge Summary. Yu et al. (2010) did not reference previous validation studies, although detection limit data were provided. Moreover, differences in assay execution were noted. Yu et al. (2010) and Ishida et al. (2011) performed HMGB1 measurements in duplicate, whereas Goggs & Letendre (2019) did not specify whether HMGB1 measurements were performed in duplicate. This variation in reporting limits comparison of assay procedures across studies, which may reduce result reliability. Specimen storage conditions also varied between studies, potentially affecting HMGB1 stability. These factors underscore the importance of both standardised measurement timing and validated assay selection, as highlighted by Matsuura et al. (2023), which found that early inhibition of HMGB1 improved survival outcomes – emphasising the critical role of timing and methodological consistency in HMGB1-related research.

Variations in breed and methodology limit the validity of cross-study comparisons. The potential for variations in prior treatments, as well as differences in the duration of illness before enrolment, likely contributed to inconsistencies in the study’s findings. These factors, including variations in disease severity, treatment protocols, and the specific demographics of dogs treated at veterinary referral hospitals, may have limited the applicability of the results to the PICO question by affecting the generalisability of the findings to a broader population of canine patients with SIRS. Methodological differences were considered across the studies, with varying outcome measures and comparisons employed. Goggs & Letendre (2019) grouped patients by non-infectious SIRS, infectious SIRS, and healthy controls, directly comparing these three groups. Yu et al. (2010) however only studied SIRS groups, comparing survivors to non-survivors, with a control group that was not directly analysed or compared to the study groups.

Ishida et al. (2011) conducted the study with the largest sample size, with a diverse range of 307 dogs with differing breeds in the study group. They also included 36 healthy Beagle dogs (15 female and 21 male) as a control group. The diseased dogs were categorised into ten groups: tumours (77/307), haematological diseases (53/307), hepatobiliary and pancreatic diseases (45/307), gastrointestinal diseases (41/307), endocrine diseases (20/307), neuromuscular diseases (19/307), skin diseases (15/307), respiratory diseases (14/307), cardiovascular diseases (11/307), and joint diseases (8/307).

Yu et al., (2010) took a similar approach, categorising diseases into groups such as trauma (n = 4), pyometra (n = 3), canine parvoviral enteritis (n = 3), heart failure (n = 2), peritonitis due to gastrointestinal tract perforation (n = 2), and other single case categories.

While Ishida et al. (2011) focuses specifically on HMGB1 as a marker of mortality in SIRS patients, it’s important to note that Goggs & Letendre (2019) did not provide a clear description of the aetiologies within their SIRS groups, which makes it more difficult to assess whether certain subtypes of SIRS were overrepresented, potentially skewing the impact of specific subgroups on the overall findings. Yu et al. (2010) included dogs representing 13 different breeds following exclusion criteria, although the individual breeds were not specified. In contrast, Goggs & Letendre (2019) provided more detailed breed information, including six Labrador Retrievers, six Staffordshire Bull Terriers, four English Bulldogs, and four mixed-breed dogs. Ishida et al. (2011) did not specify the breeds of the dogs included in the study.

The median age of the dogs also varied across studies. Ishida et al. (2011) had a median age of 8 years, the median age for Goggs & Letendre (2019) was 5.5 years and Yu et al. (2010) had a median age of 5 years. Goggs & Letendre (2019) found that age, weight, and proportion of males versus females were not significantly different between the three study groups. In contrast, Ishida et al. (2011) and Yu et al. (2010) reported age and sex distributions but did not stratify or adjust the association between HMGB1 and outcome according to age, sex, or body weight, limiting assessment of these factors as potential confounders. In particular, neither study evaluated whether the association between HMGB1 and outcome differed between young, adult, and geriatric dogs, despite evidence that inflammatory responses may vary with age (Dall’Ara, 2003; Day, 2010; Pereira et al., 2019). Overall, differences in breed and age profiles between studies limit direct comparison and the strength of the conclusions, as breed differences in inflammatory responses, disease pathogenesis, and breed-specific disease predispositions could further contribute to variability in outcomes across studies (Alexander et al., 2017; Faldyna et al., 2001).

Diagnostic limitations restricted the strength of evidence provided by all studies. Inclusion conditions for all the studies was based on having ≥ 2 SIRS criteria based on a set of criteria originally developed in Hauptman et al. (1997) and used by various other studies on SIRS (Brady & Otto, 2001; de Laforcade et al., 2003). The set of criteria developed to diagnose SIRS has been criticised to lack specificity, carrying a high risk of false positives, which is a significant limitation across the studies appraised (Hauptman et al., 1997; Ngwenyama, 2021). Goggs & Letendre (2019) also documented positive bacterial infections to distinguish between non-infectious and infectious SIRS. Interestingly, all other studies did not make this distinction and made groupings based only on non-SIRS and SIRS criteria. The appraised studies primarily assessed outcomes during the initial hospitalisation, with Yu et al. (2010) and Goggs & Letendre (2019) following dogs until hospital discharge. Ishida et al. (2011) did not report a defined long-term follow-up period, limiting assessment of outcomes beyond the initial study period. However, in the context of acute conditions like SIRS, where patients either recover or succumb to the illness within a relatively short period, a lengthy follow-up or longitudinal study might not be necessary to establish the association between serum HMGB1 levels and mortality. The mortality rate may have been assessed at different time points across studies. This variation in timing creates challenges in directly comparing the outcomes between studies.

A key methodological limitation across all three studies is the omission of data transformation prior to non-normality testing. Transforming parametric data would have maximised the information of the analysis while upholding the assumption of normality.

Yu et al. (2010) employed a Mann–Whitney U test instead of data transformation and parametric tests, which may have limited the statistical power of their analysis by relying on non-parametric methods that do not assume a normal distribution. This is due to the fact that Mann–Whitney U test is employed for small populations or skewed data. They also used a T-test in their graphical analyses, although this is not outlined in the methods section. This choice could lead to an incomplete understanding of the relationship between HMGB1 levels and mortality outcomes, necessitating a more cautious interpretation of the findings. Nevertheless, the Mann–Whitney test yielded robust statistical significance. Similarly, Goggs & Letendre (2019) proceeded directly to the Mann–Whitney test without addressing data transformation. They did, however, incorporate multivariable logistic regression, which independently assessed mortality in relation to biomarkers.

Ishida et al. (2011) also did not employ data transformation and conducted Mann–Whitney testing. While they employed a Fisher’s exact test to explore associations between variables, they did not utilise logistic regression to examine the size or direction of these associations. Notably, the study referenced specific ranges for HMGB1 but did not clearly detail how these were derived in the methods section, raising concerns about transparency. For instance, patients with HMGB1 levels above 5.46 ng/ml are 3.8 times more likely to die at discharge compared to those with levels below 5.46 ng/ml (P < 0.01).

The overall strength of evidence is of low quality. There is evidence to support the assertion that in patients with SIRS, high levels of HMGB1 and mortality are correlated (Ishida et al., 2011; Yu et al., 2010). Whilst Goggs & Letendre (2019) did not find a statistical correlation between mortality and HMGB1 concentrations, they were better able to differentiate non-infectious SIRS and infectious SIRS and from this determined that HMGB1 was significantly greater in dogs with infectious SIRS compared to the baseline of healthy dogs.

Although two of the three studies (Ishida et al., 2011; Yu et al., 2010) identified a correlation between high serum HMGB1 levels in SIRS patients and mortality, definitive conclusions remain elusive. While they provide valuable insights into the link between HMGB1 and infectious SIRS, and its potential as a mortality predictor, the current studies have limitations that affect the broader applicability of HMGB1 levels in infectious SIRS. Small sample sizes, heterogeneous sample populations, inconsistent composite outcomes and groupings, and varying statistical methods constrain the generalisability and interpretability of the findings. Future research on HMGB1 levels in infectious SIRS patients could benefit from larger, multi-centre observational studies that focus on a more homogeneous patient demographic and similar illness subgroupings. However, it is essential to balance this homogeneity with the need for a representative sample; while narrowing the patient population may enhance the clarity of findings within a specific group, it could limit the applicability of the results to the broader population of canine patients with SIRS. Therefore, carefully selecting which groups to include or exclude will be crucial to ensuring the findings are both meaningful and generalisable. Future research on HMGB1 levels in SIRS patients may include larger, multi-centre observational studies with a focus on a more homogenous patient demographic and similar illness subgroupings. Furthermore, studies where samples are taken at a certain time point post onset of illness and potentially follow trends in HMGB1 throughout the disease course may be of greater value. There is weak evidence supporting a correlation between HMGB1 levels and mortality in infectious SIRS cases; only one study (Goggs & Letendre, 2019) specifically investigates this relationship.

## Methodology

**Search Strategy:** 

Databases searched and dates covered:	CAB Abstracts on OVID platform covering 1973 to June 3 2025PubMed via the NCBI website covering 1910 to June 3 2025
Search strategy:	CAB Abstracts: (dog OR canine OR puppy) (septic systemic inflammatory response syndrome OR SIRS OR) (HMGB1 OR "high mobility group box 1") (mortality OR death OR survival OR outcome) 1 and 2 and 3 and 4 PubMed: (dog OR canine OR puppy) (septic systemic inflammatory response syndrome OR SIRS) (HMGB1 OR "high mobility group box 1") (mortality OR death OR survival OR outcome) 1 and 2 and 3 and 4
Dates searches performed:	3 June 2025

**Exclusion / Inclusion Criteria:** 

Exclusion:	Not relevant to parameters of the PICO (only including non-infectious SIRS, not evaluating SIRS mortality outcomes in relation to HMGB1, only including sepsis, biomarkers outside of HMGB1). Studies limited to a single underlying disease process (e.g. pancreatitis) rather than evaluating a general SIRS population. Case studies. Study summary or article. Systematic review, single case reports. Duplicate. Studies without a defined SIRS population (e.g. those looking only at sepsis without SIRS criteria).
Inclusion:	Canine subjects. Peer reviewed publication. English language.

**Search Outcome:** 

Database	Number of results	Excluded – case study	Excluded – non-dog	Excluded – not evaluating effects	Excluded – not evaluating in hospital setting or confinement	Excluded – article	Excluded – systematic review	Total relevant papers
Cab Abstracts	1	0	0	0	0	0	0	1
PubMed	4	0	0	0	0	0	1	3
Total relevant papers when duplicates removed								3

## Acknowledgements

Thank you to Dr Samantha Livingstone for her guidance and contribution of knowledge.

## ORCiD

Hannah Abrahams: 0009 0007 5221 5723

## Conflict of Interest

The authors declare no conflicts of interest.

## Disclaimer

Knowledge Summaries are a peer-reviewed article type which aims to answer a clinical question based on the best available current evidence. It does not override the responsibility of the practitioner. Informed decisions should be made by considering such factors as individual clinical expertise and judgement along with patient’s circumstances and owners’ values. Knowledge Summaries are a resource to help inform and any opinions expressed within the Knowledge Summaries are the author's own and do not necessarily reflect the view of the RCVS Knowledge. Authors are responsible for the accuracy of the content. While the Editor and Publisher believe that all content herein are in accord with current recommendations and practice at the time of publication, they accept no legal responsibility for any errors or omissions, and make no warranty, express or implied, with respect to material contained within. For further information please refer to our Terms of Use.
